# Comprehensive analysis of the proximity-dependent nuclear interactome for the oncoprotein NOTCH1 in live cells

**DOI:** 10.1016/j.jbc.2023.105522

**Published:** 2023-12-01

**Authors:** Haydee M. Torres, Fang Fang, Danielle G. May, Paige Bosshardt, Leetoria Hinojosa, Kyle J. Roux, Jianning Tao

**Affiliations:** 1 Cancer Biology & Immunotherapies Group, Sanford Research, Sioux Falls, South Dakota, USA; 2 Department of Chemistry and Biochemistry, South Dakota State University, Brookings, South Dakota, USA; 3 Enabling Technologies Group, Sanford Research, Sioux Falls, South Dakota, USA; 4 Department of Pediatrics, Sanford School of Medicine, University of South Dakota, Sioux Falls, South Dakota, USA

**Keywords:** Notch pathway, cancer, protein-protein interaction, HDAC1, Proteomics, NOTCH1, BioID, NICD, network analysis

## Abstract

Notch signaling plays a critical role in cell fate decisions in all cell types. Furthermore, gain-of-function mutations in NOTCH1 have been uncovered in many human cancers. Disruption of Notch signaling has recently emerged as an attractive disease treatment strategy. However, the nuclear interaction landscape of the oncoprotein NOTCH1 remains largely unexplored. We therefore employed here a proximity-dependent biotin identification approach to identify *in vivo* protein associations with the nuclear Notch1 intracellular domain in live cells. We identified a large set of previously reported and unreported proteins that associate with NOTCH1, including general transcription and elongation factors, DNA repair and replication factors, coactivators, corepressors, and components of the NuRD and SWI/SNF chromatin remodeling complexes. We also found that Notch1 intracellular domain associates with protein modifiers and components of other signaling pathways that may influence Notch signal transduction and protein stability such as USP7. We further validated the interaction of NOTCH1 with histone deacetylase 1 or GATAD2B using protein network analysis, proximity-based ligation, *in vivo* cross-linking and coimmunoprecipitation assays in several Notch-addicted cancer cell lines. Through data mining, we also revealed potential drug targets for the inhibition of Notch signaling. Collectively, these results provide a valuable resource to uncover the mechanisms that fine-tune Notch signaling in tumorigenesis and inform therapeutic targets for Notch-addicted tumors.

Notch signaling is an evolutionarily conserved intercellular communication mechanism across invertebrates and vertebrates that regulate cell fate determination, proliferation, differentiation, and death programs (1). In normal mammalian cells, there are four Notch receptors (named Notch1-Notch4), which are single pass transmembrane proteins that can be activated by physical interaction with a transmembrane ligand on juxtaposed cells, resulting in the ligand-induced proteolytic cleavages of the receptor and the release of the COOH-terminal portion of the Notch intracellular domain (NICD) (2). The NICD enters the cell nucleus, binds to a transcription factor recombination signal binding protein for immunoglobulin kappa J region (RBPJ) (also known as CBF1/Suppressor of Hairless/Lag-1 [CSL]), and then interacts with a coactivator mastermind-like protein 1 (MAML1) to form a stable ternary complex NICD/RBPJ/MAML1, which serves as a required platform for recruiting auxiliary coregulators to assemble a larger Notch transcriptional complex to activate downstream target genes (3, 4). Nevertheless, the short half-life of NICD and the lack of amplification in stoichiometric signal transduction render the Notch pathway sensitive to precise regulation influenced by dose, duration, and the epigenetic context of the signal (5). Moreover, how the NICD precisely activates transcription and interacts with its partners in the nucleus remains incompletely understood.

Derailed Notch signaling is associated with congenital, late-onset disorders and cancer (6). Recent genomic studies have identified cancer-specific gain- or loss-of-function mutations in Notch genes and implicated distinct roles for Notch, ranging from oncogenic to tumor suppressive, depending on cancer type (7). The oncogenic role of strong gain-of-function mutations in NOTCH1 has been experimentally demonstrated in T-cell acute lymphoblastic leukemia/lymphoma (T-ALL) (1). These mutations occur in the juxtamembrane negative regulatory region and/or the C-terminal PEST degron domain of the NOTCH1 receptor, leading to the constitutive generation of high levels of NICD and ligand-independent Notch activation (8). Similarly, strong gain-of-function mutations in NOTCH1 have been found in several solid tumors, including breast cancer and adenoid cystic carcinoma (9, 10, 11, 12). Importantly, deregulated Notch signals with a long duration in tumor cells may lead to the activation of a large set of target genes, perhaps through interacting with auxiliary coregulators and protein modifiers that are not recruited by the WT Notch protein under normal conditions (13, 14).

Given its role in cancer and its therapeutic applications in a variety of disorders, it is valuable to identify the protein components of the NOTCH1 transcription complex and its functional protein-protein association network. In the current study, we investigated a nuclear interactome for NOTCH1 oncoprotein in live cells using proximity-dependent biotin identification (BioID), which was developed to overcome barriers imposed by conventional screening methods for protein-protein interactions (PPIs) (15). The BioID method can be used to characterize the nuclear environment occupied by an oncogenic protein such as NOTCH1 and a history of its PPIs in living cells (16). Here, we fused a second-generation mutant biotin ligase (BioID2) to the “bait” protein NICD, which can release biotinoyl-AMP into the proximal environment to covalently label lysine residues of the bait within ∼ 10 nm. These biotinylated proteins including poorly soluble nuclear chromatin cofactors can be selectively isolated using harsh lysis conditions, then captured by streptavidin affinity, and identified by mass spectrometry (MS) (17). Since the average globular protein diameter is less than 10 nm, these candidates identified by the BioID method favor direct binding partners and components of protein complexes in which the bait lodges (18). Due to its applicability to weak/transient PPIs and insoluble proteins, the BioID method has rapidly become widely used to define the composition of many different protein complexes and PPIs in different cellular compartments, including the identification of transcription factor complexes in the nucleus (19, 20, 21). Hence, the nuclear interactome of Notch1 generated in this study will benefit further investigations of the molecular mechanisms of NOTCH1 functions and regulation that govern Notch transcriptional activity in normal and cancer cells.

## Results

### Identification of NOTCH1 proximal interacting proteins using BioID in HEK293 cells

**Figure 1:**
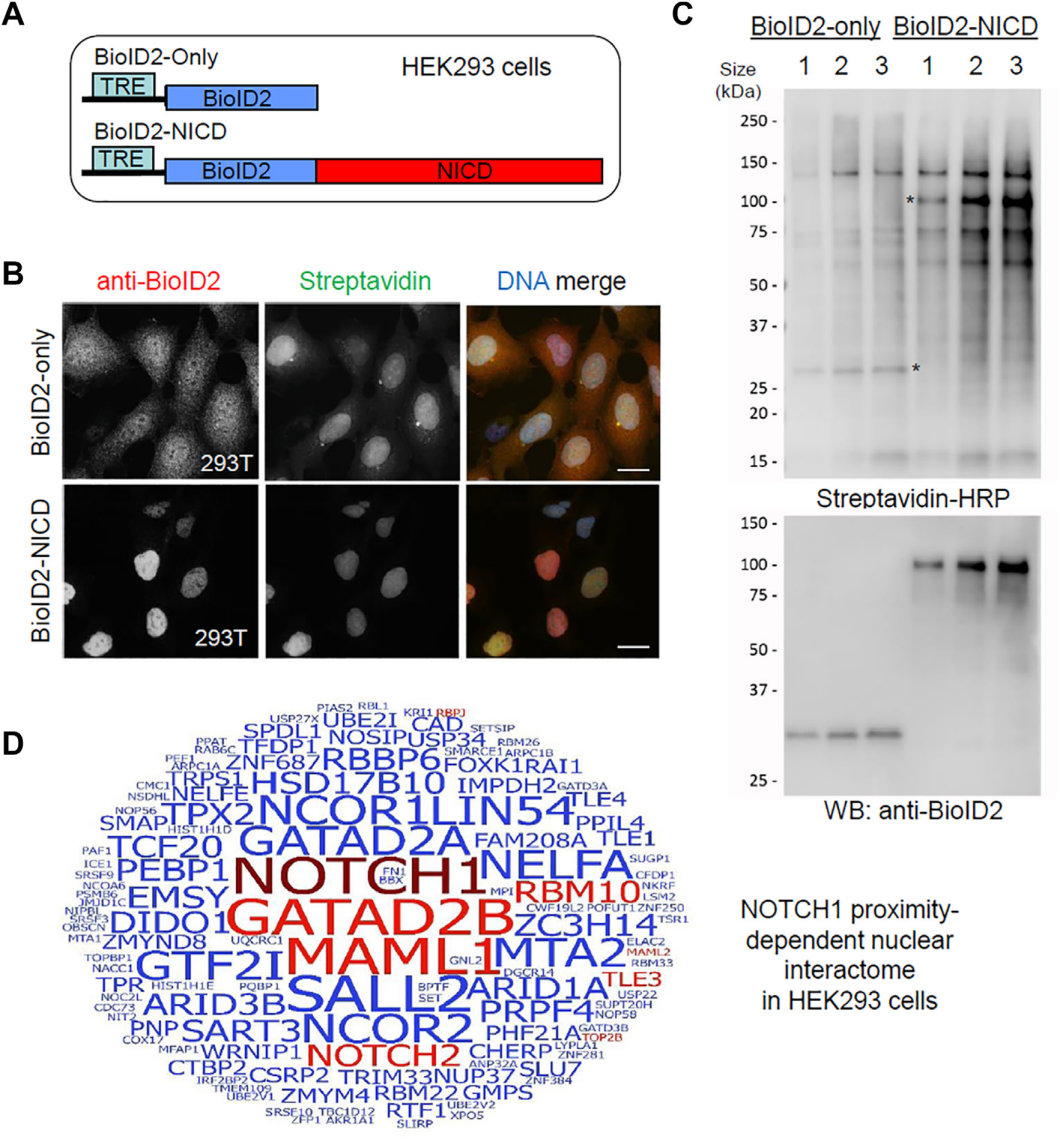
**Identification of Notch1 proximity-dependent interacting proteins in HEK293 cells.***A*, the schematic of doxycycline-inducible constructs under the tetracycline response element (TRE) promoter. Notch1 intracellular domain (NICD) was fused to C terminus of the promiscuous biotin ligase BioID2. *B*, immunofluorescence analyses of HEK293 cells stably expressing fusion protein detected with anti-BioID2 (*red*) and promiscuous biotinylation detected with fluorescently labeled streptavidin (*green*) following the addition of exogenous doxycycline and biotin. The scale bar represents 20 μm. *C*, Western blot (WB) analysis of biotinylated proteins detected with streptavidin-HRP. *Asterisks* indicate the location of the BioID2-fusion protein (detected with anti-BioID2). Each lane represents a single biological replicate used in the BioID study. *D*, word cloud diagram depicting Notch1 interacting proteins identified by mass spectrometry (MS). The size of each protein name is proportional to the number of identified total MS/MS spectral counts for each protein. Candidate proteins previously reported listed as Notch1 interactors in the BioGRID database are shown in *red*, and previously unreported candidate Notch1 interactors are shown in *blue*. BioID, biotin identification; HRP, horseradish peroxidase.

Strong gain-of-function mutations in NOTCH1 leading to ligand-independent accumulation of NICD in the nucleus is a common event in human cancers, and expression of a truncated NICD in many different cell types in mice can drive spontaneous tumor formation (7, 22, 23, 24). Therefore, understanding proximal interacting proteins in NOTCH1 complexes will be instrumental to define its role in tumorigenesis at the molecular level. To identify such proteins, we employed a second generation BioID system (Fig. S1), which utilizes a modified, smaller *Aquifex aeolicus* biotin ligase (BirA∗- R40G), called BioID2 (18). The BioID2 was fused to the N terminus of the truncated NICD protein and BioID2-only was used as a control (Fig. 1*A*). In order to minimize artificial interactions and protein instability caused by overexpression of the bait proteins, we utilized a doxycycline-inducible system in human HEK293 stable cell line allowing moderate and inducible bait protein expression (Fig. S2). We chose to use HEK293 cells because it is a highly transfectable cell line and has been used successfully by us and others for large-scale BioID pull-downs of diverse types of bait proteins (15, 21, 25, 26). Each HEK293 cell line was validated by immunofluorescence (IF) (Fig. 1*B*) and Western blot (WB) (Fig. 1*C*) for fusion-protein expression and biotinylation, revealing that their expression levels in nucleus and overall biotinylation were comparable. As expected, the BioID2 protein was localized to both the cytoplasm and the nucleus, whereas BioID2-NICD was localized to the nucleus only (Fig. 1*B*). Each cell line was processed in biological triplicate and subjected to affinity purification of biotinylated proteins for identification of proximal prey proteins *via* semiquantitative tandem MS (Fig. S2). Peptide and protein identification, intensity quantification, and data analysis were performed to identify and summarize proximal proteins in Table S1, *A* and *B*. Overall, a total of 133 BioID candidate interaction proteins were identified (Table S1*C*). The number and intensity of the recovered peptides indicate that MAML1 and GATAD2B are the closest components of the Notch activation complex, and their names are highlighted in larger font in Figure 1*D*. Other major Notch pathway components such as RBPJ, MAML2, and NOTCH2 were also recovered. Prominently, from this list, 10 of the proteins were previously known interacting proteins (as determined from BioGRID (27)) and are highlighted (red letters) in Figure 1*D*. Altogether, these results validate our BioID-identification strategy.

### Identification of NOTCH1 proximal interacting proteins using BioID in NIH3T3 cells

**Figure 2:**
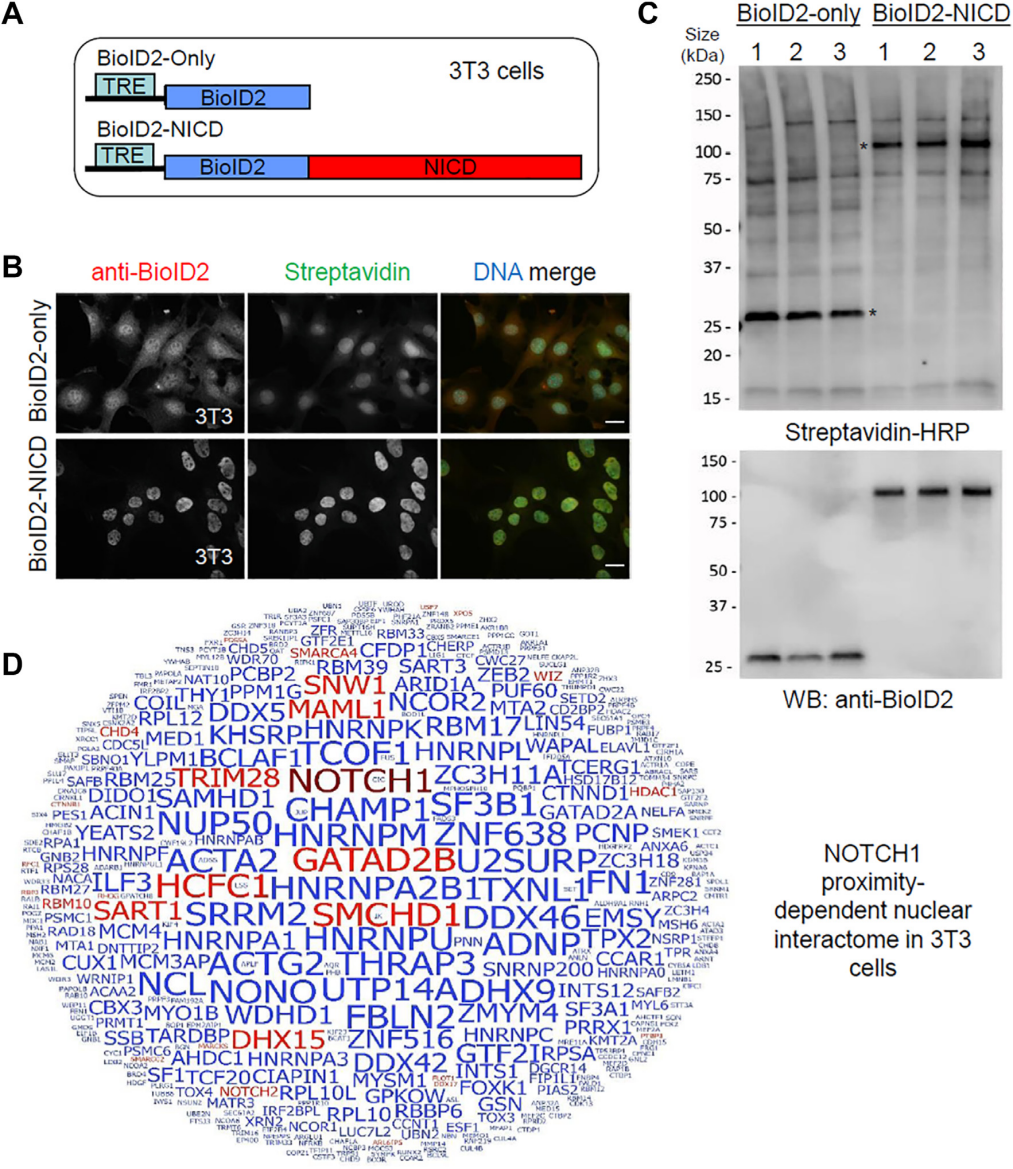
**Identification of Notch1 proximity-dependent interacting proteins in 3T3 cells.***A*, schematic of doxycycline-inducible constructs under the tetracycline response element (TRE) promoter for BioID2-only and BioID2-NICD. *B*, immunofluorescence analyses of 3T3 cells stably expressing fusion protein detected with anti-BioID2 (*red*) and promiscuous biotinylation detected with fluorescently labeled streptavidin (*green*) following the addition of exogenous doxycycline and biotin. The scale bar represents 20 μm. *C*, Western blot (WB) analysis of biotinylated proteins detected with streptavidin-HRP. *Asterisks* indicate the location of the BioID2-fusion protein (detected with anti-BioID2). Each lane represents a single biological replicate used in the BioID study. *D*, word cloud diagram depicting Notch1 interacting proteins identified by mass spectrometry (MS). The size of each protein name is proportional to the number of identified total MS/MS spectral counts for each protein. Candidate proteins previously reported listed as Notch1 interactors in the BioGRID database are shown in *red*, and previously unreported candidate Notch1 interactors are shown in *blue*. BioID, biotin identification; HRP, horseradish peroxidase.

**Figure 3:**
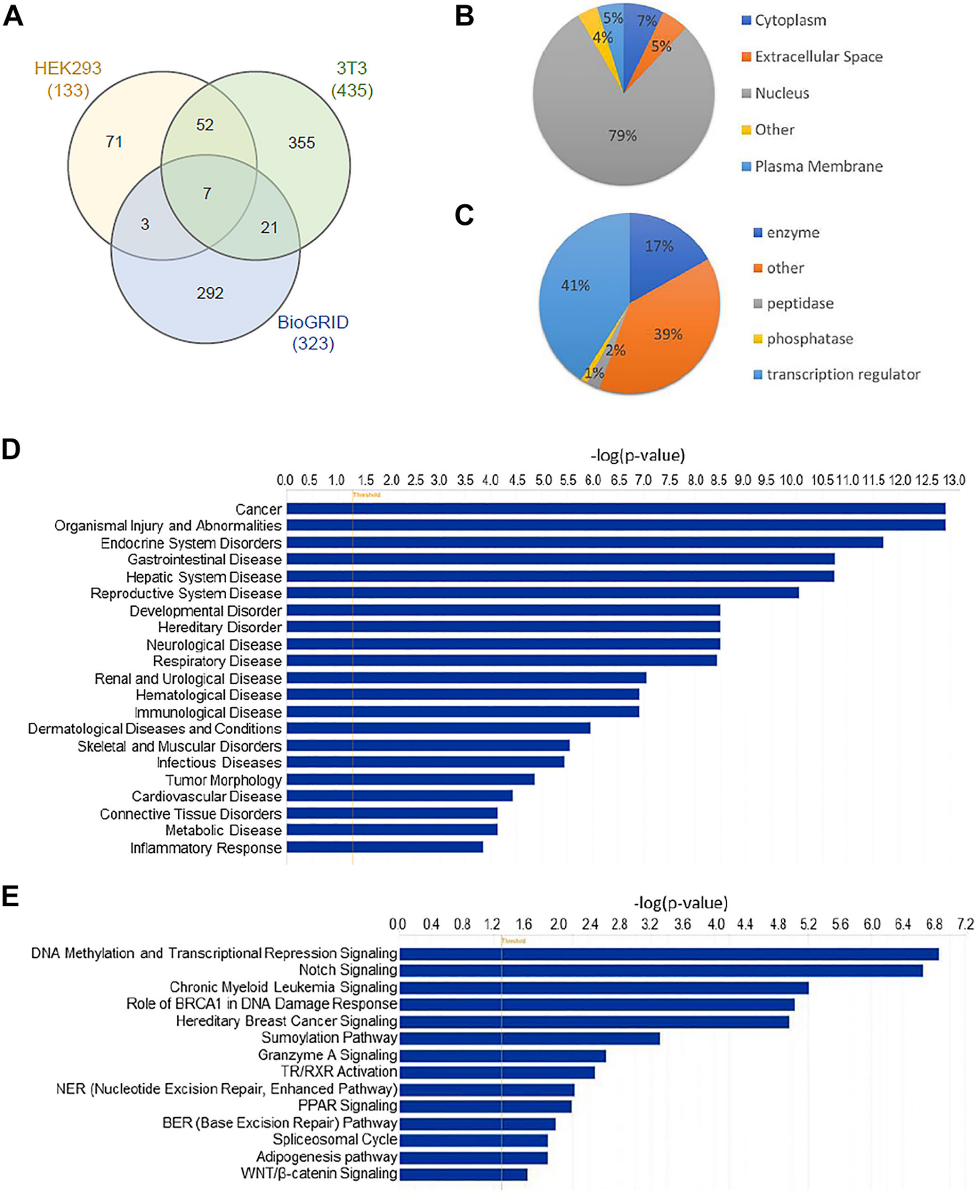
**Functional and pathway analysis of Notch1 core proximal proteins.***A*, Venn diagram depicting the number of BioID candidate interaction proteins among HEK293, 3T3, and BioGRID. *B*, protein localization of core proximal proteins. *C*, molecular function of core proximal proteins. *D*, ingenuity pathways analysis (IPA) of disease categories associated with core proximal proteins. *E*, IPA analysis of canonical signaling pathways associated with core proximal proteins. BioID, biotin identification.

Crossover studies across different cell types and different species suggest that Notch1 may have a dynamic set of proximal interacting proteins that activate common and diverse target genes under different circumstances (28, 29, 30). To understand more about those Notch1-proximal proteins in different cell types, we performed BioID studies in the mouse embryonic fibroblast cell line NIH3T3 (also called 3T3) (Figs. 2*A* and S2). Each 3T3 cell line was validated by IF (Fig. 2*B*) and WB (Fig. 2*C*) for fusion-protein expression and biotinylation, revealing their expression levels in the nucleus and that overall biotinylation was comparable among three biological replicates. Raw and analyzed proteomic data were summarized in Table S2, *A* and *B*. A total of 435 BioID candidate interaction proteins were identified (Table S2*C*). The number and intensity of the recovered peptides indicated that MAML1 and GATA2B are the closest components of the Notch activation complex among many known NOTCH1 interactors, including SMCHD1, HCFC1, and SNW1. Other major Notch pathway components such as RBPJ and NOTCH2 were also recovered. Prominently, from this list, 28 of the proteins were previously known interacting proteins of NOTCH1 (as determined from BioGRID (27)) and are highlighted (red letters) in Figure 2*D*. Moreover, 59 common proximal interacting proteins were shared between HEK293 and 3T3 cells (Fig. 3*A*). Altogether, these results validate our BioID-identification strategy in a second cell type. However, the majority of proteins identified in each cell type are distinct, supporting the reasoning that Notch has complex and dynamic relationships with various nuclear cofactors in any given context (7).

### Functional characterization of NOTCH1 core-proximal proteins reveals key pathways involved in human cancer and other diseases

Constitutively active Notch in tumor cells may generate stronger signals of longer duration, resulting in gain-of-function interactions that do not exist under physiological conditions. Our BioID study attempted to mimic those conditions in tumor cells to capture previously unidentified proximal interacting proteins of Notch1. Furthermore, the common oncogenic role of NICD in various cell types suggests that NOTCH1 may regulate some common transcriptional programs and pathways through a shared set of common proximal proteins. This prompted us to focus on analyzing those common proteins identified in at least two cell types or studies, including those identified in HEK293 and 3T3, HEK293 and BioGRID, and 3T3 and BioGRID (Fig. 3*A*). A total of 83 common Notch1 proximal interactors, referred to as core-proximal proteins in this study, were identified, and summarized in Table S3*A*. Gene ontology analysis of these 83 core-proximal proteins revealed that the majority (79%) of them were localized in the nucleus, consistent with the nature of the NICD protein (Fig. 3*B*). Notably, we identified the plasma membrane proteins, FLOT1 and MARCKS that can, like NOTCH1 and NOTCH2, also translocate into the nucleus (31, 32). Likewise, candidate proteins listed in the cytoplasm, extracellular space and elsewhere may be able to translocate into the nucleus to interact with NICD (Table S3*B*). For example, AKR1A1, RAI1, CFDP1, and RBM33 have been reported to have functional roles in the nucleus (33, 34, 35, 36). Protein function analysis showed that many of the proximal proteins have roles in transcription regulation and enzyme processes (Fig. 3*C*). Approximately 39% of the core-proximal proteins have other functions (Table S3*B*). Not surprisingly, the disease most associated with NOTCH1 core-proximal proteins was predicted to be cancer (Fig. 3*D*). Other diseases are linked to nearly every major system of our body and inflammatory response, consistent with the important role of Notch1 signaling in development, homeostasis, and viral infection (Table S3*C*). Ingenuity pathway analysis (IPA) predicted 37 canonical signaling pathways for Notch1 core-proximal proteins (Table S3*D*). “Notch signaling” and “DNA methylation and transcriptional repression signaling” were the top two most prominent of the predicted signaling pathways for Notch1 proximal proteins followed by many interesting pathways such as sumoylation, granzyme A, TR/RXR, BER, adipogenesis, and WNT/β-catenin signaling pathways (Fig. 3*E*). However, cross talk between Notch signaling and other pathways remains to be determined.

### Protein networks of NICD proximal interactors reveal key regulators and modular complexes that contribute to pleiotropic Notch functions

**Figure 4:**
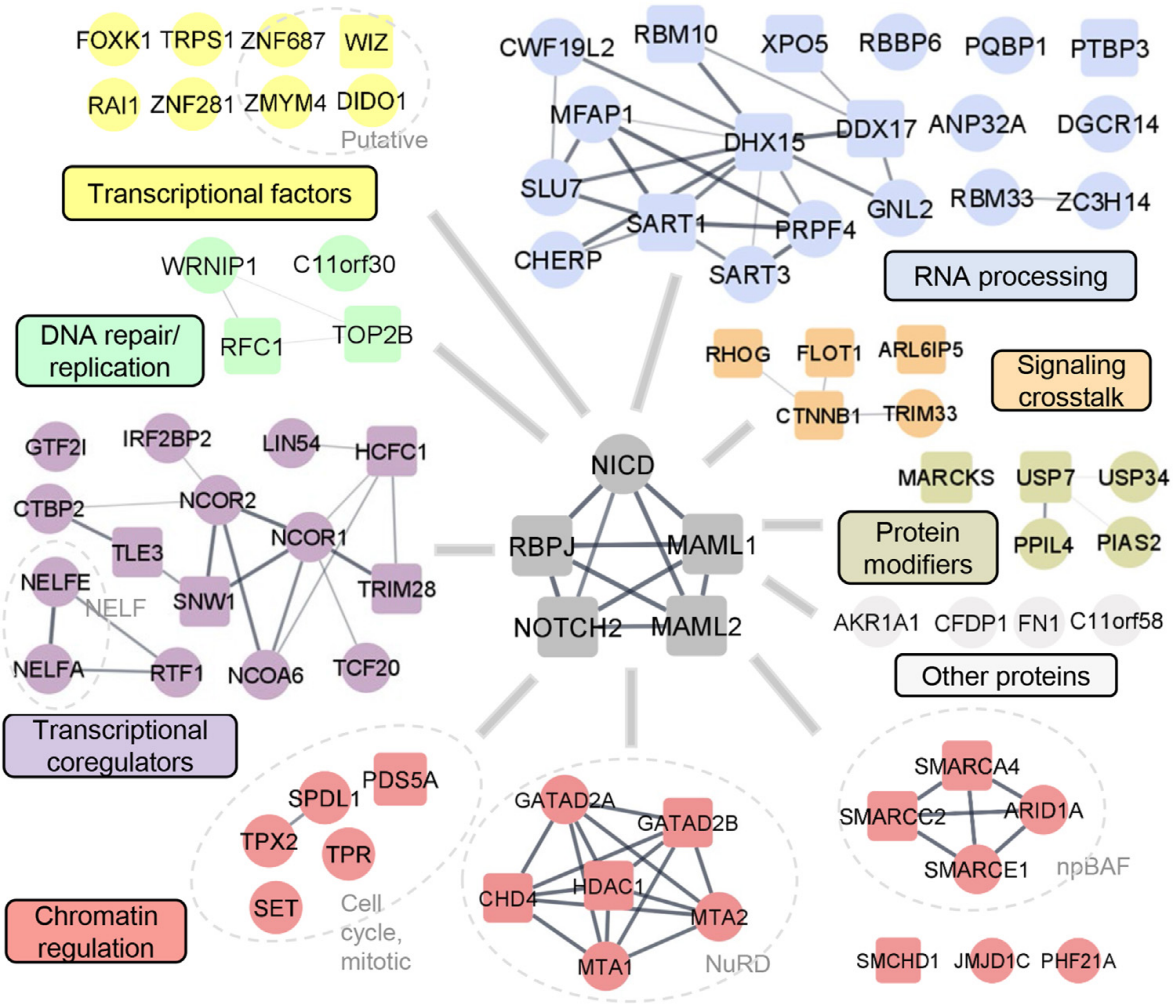
**Protein networks of NICD proximal interactors and their biological functions**. NICD-proximal proteins (described in Table S4*B*) were grouped into known protein classes or complexes as annotated in STRING and visualized using Cytoscape. Previously published NOTCH1 binding partners are represented by *square nodes*, and novel interactors are represented by *round nodes*. For clarity, interclass (or intercomplex) edges are not shown. NICD, Notch1 intracellular domain.

To understand the functional and biochemical relationships between the identified interactors of NICD, we conducted PPI network analysis of 83 core-proximal proteins (Table S4*A*). We observed that most of the identified NICD-proximal proteins are significantly associated (Fig. S3) and are related to several functional classes of proteins (Fig. 4). These include chromatin-modifying interactors as subunits of chromatin-remodeling modular complexes SWI/SNF (*e.g.* npBAF) and nucleosome remodeling and deacetylating (NuRD), and complex subunits in regulation of the mitotic cell cycle. Some proteins have been previously reported to be physically associated with NOTCH1 such as histone deacetylase (HDAC)1, GARAD2B, CHD4, SMARCA4, SMARCC2, PDS5A, and SMCHD1 (14). PHF21A (a subunit of the corepressor of repressor element-1 silencing transcription deacetylase complex (CoREST) and BHC corepressor complex that acts by deacetylating and demethylating specific sites on histones) and the histone demethylase Jumonji domain containing 1C (JMJD1C) were also detected (37, 38, 39).

Notably, among NICD core-proximal proteins we found abundant transcriptional regulatory proteins that regulate gene expression at stages of transcriptional initiation and elongation (Table S4*B*). These include well-characterized coactivators, such as NCOA6 and SNW1, and corepressors, such as NCOR1 and NCOR2, subunits of the negative elongation factor complex, as well as known (FOXK1, TRPS1, RAI1, and ZNF281) or putative transcription factors (ZNF687, WIZ, ZMYM4, and DIDO1). For example, direct genomic targets of TCF20 and TRPS1, a lineage-specific transcription factor, have remained poorly characterized because it may affect transcription positively or negatively as a component in various chromatin complexes depending on the cell types (40, 41, 42). Our NICD core-proximal interactors also comprise proteins involved in DNA repair and replication including WRNIP1, replication factor C (RFC)1, TOP2B, and C11orf30 (also known as EMSY). Previous studies have reported that RFC1 and TOP2B are physically associated with NICD, and NICD is also functionally associated with the RFC complex (14, 43).

Our data also highlight interactions with proteins involved in RNA processing, such as RNA-binding proteins (RBM10 and XPO5) and subunits of spliceosomal complex (DHX15 and DDX17). Moreover, interactions between NICD and components of other signaling pathways, such as the Rho GTPase family member RhoG and the WNT canonical signaling central mediator β-catenin (CTNNB1), were also recovered. In agreement with others’ reports (14, 21, 44, 45), we also uncovered interactions with protein-modifiers (MARCKS, USP7, USP34, PPIL4, and PIAS2). For example, USP7 has previously been implicated in the regulation of NOTCH1 protein stability and activation (44, 45). Hence, NICD interacts with diverse complexes and regulators that may reflect pleiotropic Notch functions.

### Essential interactors in the NOTCH1 protein network as potential drug targets

**Figure 5:**
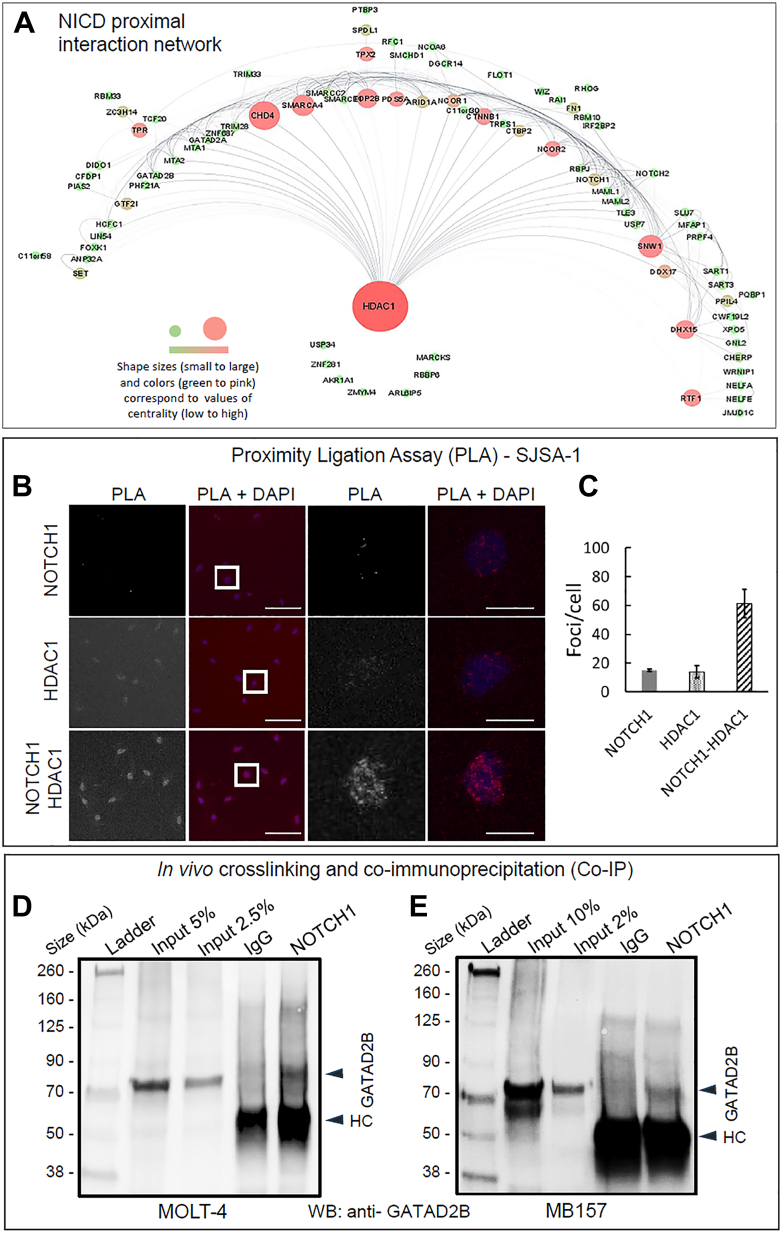
**High-betweenness protein nodes in the NICD proximal interaction network and *in vivo* interactions between endogenous proteins in Notch-addicted cancer cells.***A*, illustration of betweenness centrality nodes in NICD proximal interaction network. The betweenness centrality values are calculated using the NetworkAnalyzer Cytoscape plugin. Networks were visualized using yFiles Radial Layout in Cytoscape, whereby larger shape sizes and darker colors correspond to higher values of betweenness centrality. *B*, validation of the NOTCH1-HDAC1 interaction in human SJSA-1 osteosarcoma cells by the proximity ligation assay. Representative images of PLA signal and nuclear staining (DAPI) showing cells probed with anti-Notch1 alone (*top row*), anti-HDAC1 alone (*middle row*) or both antibodies (*bottom row*). The scale bars at low magnification (*two left panels*) represent 100 μm, and the scale bars with high magnification (*two right panels*) represent 20 μm. *C*, quantification of mean PLA per foci per cell, with standard deviation; n = 3. *D*, Western blot (WB) analysis of immunoprecipitates (IP: NOTCH1 and control IgG antibodies) and nuclear extracts from MOLT-4 leukemia cells cross-linked with DSP showing endogenous protein GATAD2B coimmunoprecipitated with NOTCH1. Both immunoprecipitating and immunoblotting antibodies were raised in rabbits. *Arrow heads* indicate IgG heavy chain (HC) and GATAD2B. The ladder represents the molecular weight (kDa) of the protein size. The input lanes represent the initial input in the experiment (5% and 2.5%, respectively). *E*, WB analysis of immunoprecipitates (IP: NOTCH1 and control IgG antibodies) and nuclear extracts from MB157 breast cancer cells cross-linked with DSP showing endogenous protein GATAD2B coimmunoprecipitated with NOTCH1. The input lanes represent the initial input in the experiment (10% and 2%, respectively). DAPI, 4′,6-diamidino-2-phenylindole; DSP, 3,3′-Dithiodipropionic acid di(N-hydroxysuccinimide ester; IgG, immunoglobulin G; NICD, Notch1 intracellular domain; PLA, proximity-based ligation assay.

The centrality-lethality hypothesis postulates that protein nodes with higher centrality in a network are more likely to produce a lethal phenotype when removed than nodes with lower centrality (46). The essential protein nodes (or interactors) can be identified by measuring higher value of betweenness centrality (47). Here, we analyzed high-betweenness nodes in the NICD proximal protein interaction network (48). We found HDAC1 to be the central node with the highest betweenness centrality measure, followed by CHD4, SNW1, SMARCA4, and TOP2B (Fig. 5*A*). HDAC1 also has the highest closeness centrality measure in the network (Table S5*A*). This is not surprising since HDAC1 and HDAC2 (HDAC1/2) are part of numerous modular complexes that contribute approximately half of the total deacetylase activity of the 18 HDACs found in mammalian cells (49, 50). To validate interactions and physical closeness of HDAC1 and NOTCH1 proteins in cells using an additional approach, we performed *in situ* proximity-based ligation assay (PLA) in human SJSA-1 osteosarcoma cells. We found that significant and specific nuclear fluorescence signals were detected when SJSA-1 cells were probed with NOTCH1 and HDAC1 antibodies (Fig. 5*B*, bottom row, and Fig. 5*C*), whereas addition of NOTCH1 or HDAC1-specific antibodies alone produced only background levels of fluorescence (Fig. 5*B* first and second row). Independently, their PLA interaction was validated in 293T cells (Fig. S4*A*), suggesting that it is not restricted to one cell type.

To further functionally characterize newly identified interactions related to HDAC1, we applied *in vivo* chemical crosslinking and endogenous coimmunoprecipitation (Co-IP) to examine whether GATAD2B, an understudied component of the NuRD complex, can directly interact with NOTCH1 in MOLT-4 and MB157 cell lines. Cells from both present high expression of NICD protein with activating mutations of *NOTCH1* (8, 14, 30). We chose 3,3′-Dithiodipropionic acid di(N-hydroxysuccinimide ester (DSP), a homogeneous bifunctional cell-permeable cross-linker with a 12-Å (1.2 nm) spacer arm, which allows cleavage of cross-linked products and to identify labile proximity-protein interactions in live cells (51, 52, 53). Here Co-IP analysis revealed that endogenous GATAD2B was readily detectable in MOLT-4 cells (Fig. 5*D*) and MB157 cells (Fig. 5*E*). Interestingly, we also observed that endogenous DDX15 coprecipitated with endogenous RBPJ in MOLT-4 cells (Fig. S4, *B* and *C*). Unlike its family member DDX5 (54), an ATP-dependent RNA helicase, the role of DDX15 in its functional interaction with the NOTCH1 complex and in T-ALL leukemia cells is unclear. Our data suggest that HDAC1 and some other identified interacting proteins such as GATAD2B in this study may be essential partners for NOTCH1 function and may serve as important therapeutic targets in NOTCH1-dependent cancers.

On the other hand, for drug repurposing considerations, we sought to gain an unbiased understanding of how many proximal proteins of NOTCH1 in this study could be targeted by currently available drugs or agents that are Food and Drug Administration (FDA)-approved or examined in clinical trials or preclinical studies. We cross-referenced our Notch1 proximal proteins with potential therapeutic targets annotated in CLUE drug library (55) and IPA database (56). We identified a total of 10 targets, including five targets with 15 known FDA-approved drug interactions with potential for therapeutic use or testing in Notch1-dependent cancers (Fig. S5 and Table S5, *B* and *C*). For example, our data support current clinical trials and ongoing studies to treat NOTCH1-dependant T-ALL by repurposing FDA-approved HDAC inhibitor drugs, including Vorinostat, Romidepsin, Belinostat, and Panobinostat, which specifically target HDAC1 together with one or more other HDACs (57, 58). However, continued investigation is needed to validate these potential targets and the possible impact of drugs or agents on NOTCH1-dependent cancers.

## Discussion

We have applied an innovative and alternative approach to define the biochemical composition of the nuclear Notch complexes in live cells. Our BioID study has complemented previous characterization of NICD partners and PPIs using conventional low or high throughput methods such as reconstituted complex, yeast two-hybrid, and tandem affinity purification (13, 14, 59, 60, 61, 62, 63, 64, 65, 66). Moreover, our BioID findings provide a framework to further define the complex architecture and elucidate NOTCH1 regulation and mechanisms of action.

The core Notch-activation complex NICD/RBPJ/MAML1 together with a number of nuclear coregulators initiates transcription of Notch target genes, which execute Notch functions (3, 4, 7, 67). Our BioID data expand our understanding of multiple functionally relevant protein complexes involved in different steps of the transcriptional activation process such as transcriptional initiation, chromatin remodeling, elongation by RNA polymerase II, and DNA replication and repair (Fig. 4). Consistent with reports by others (Yatim *et al*., 2012), we found that the NICD interacts with components of SWI/SNF remodeling complex including SMARCA4 and SMARCC2 (also known as BRG1/BAF190 and BAF170). Moreover, we also found NICD interacting with unreported ARID1A and SMARCE1 (also known as BAF250 and BAF57). Interestingly, recurrent mutations of ARID1A and NOTCH1 have been frequently associated with many types of cancer (68, 69, 70, 71, 72). However, our study did not identify the reported coactivator AF4p12 and transcription factors such as IKAROS and RUNX1, which may have tissue-specific roles in T-ALL (14). Instead, our data revealed other unreported nuclear coregulators, including TCF20, LIN54, TRPS1, DIDO1, and RAI1 that may participate in Notch-mediated transcriptional activation by serving as upstream “pioneer” transcription factors that can bind to and open up repressed chromatin or downstream “settler” transcription factors that act through regulatory elements. Additionally, TCF20 can form a complex with RAI1 (42). Individually, *de novo* nonsense and frameshift variants of TCF20 have been reported in individuals with intellectual disability and postnatal overgrowth (73). Mutations in RAI1 are associated with Smith–Magenis syndrome, a developmental disorder characterized by mental retardation and craniofacial and skeletal abnormalities (74). Further studies are needed to better understand the functional interaction of the TCF20/RAI1 complex with the Notch-activation complex in these diseases.

It remains largely unclear how the strength of the Notch response is regulated by the relative abundance of activating and repressive complexes as well as the competition between them (4). The current view is that RBPJ is a transcriptional repressor that interacts with many nuclear corepressor proteins such as HDAC1 (75) in the absence of Notch signaling, whereas once bound to the nuclear NICD, RBPJ is converted into a transcriptional activator (7). Interestingly, our studies indicate that NICD may directly interact with components in different corepressor complexes, including NuRD (GATAD2A, GATAD2B, CHD4, HDAC1, MTA1, and MTA2), CoREST (HDAC1, CTBP2, and PHF21A) and SMRT/NCOR (NCOR1 and NCOR2). NuRD and CoREST are two of four canonical corepressor complexes containing HDAC1/2 (76), whereas in our BioID study we did not observe NICD interacting with the components of the two canonical Sin3 and MiDAC complexes. Although NuRD has long been accepted as a transcriptional repressor, more recent data have shown that it is prevalent at enhancers and promoters of active gene loci and is likely associated with active transcription (77, 78). It may be interesting to explore how repressive complexes such as NuRD interact with NOTCH1-RBPJ activating complex in the recently discovered long-range super-enhancer complexes (79, 80). The results of our PLA and Co-IP analyses suggest that HDAC1 and GATAD2B may be part of the Notch activating complex or the Notch inhibitory complex, or both (Fig. 4, *B*–*E*). Moreover, SMRT/NCOR is a corepressor complex containing HDAC3, which can positively regulate Notch signaling through controlling NICD protein acetylation and stability (81), but our study did not detect NICD-HDAC3 interaction. Instead, consistent with previous findings on the physical interaction between HDAC1 and NICD in leukemia cells (14), we validated NICD-HDAC1 interactions in SJSA-1 and 293T cells (Figs. 5, *B* and *C* and S4). SJSA-1 cells possess WT NOTCH1 but are highly sensitive to Notch inhibitors and a HDAC1 inhibitor (82, 83). On the other hand, in a *Drosophila* wing study, depletion of HDAC1 causes reduced expression of Notch and its target genes, suggesting that HDAC1 positively regulates Notch signaling by promoting Notch transcription (84). Consistent with the *Drosophila* study, our recent work and others indicate that HDAC1 function is required for the positive regulation of Notch signaling-meditated cell differentiation in vascular and bone cells (85, 86). Thus, it will be important to determine whether HDAC1 can positively regulate Notch signaling by either controlling NICD stability and/or transcription levels in the context of solid tumors.

Several histone modifiers identified in this study provided evidence supporting that histone H3 lysine (H3K) modifications play a critical role in the epigenetic regulation of Notch target genes (Fig. 4). Previous studies show that Notch activation results in the acquisition of active marks, including acetylation of H3K27 (79) and H3K56 (87) in Notch-regulated enhancer elements and trimethylation (me3) of H3K4 in the Notch-regulated gene promoters (88). In corresponding regions, repressive marks such as H3K27Me3 are lost (89). In T-ALL cells, Yatim *et al*. found two histone demethylases: LSD1/KDM1A functions as a corepressor when associated with CSL-repressor complex (*via* removing active mark H3K4me2) and as a NOTCH1 coactivator upon Notch activation (*via* removing repressive mark H3K9me2); PHF8/KDM7B may promote epigenetic modifications by removing repressive mark H3K27me2 (14). In our study, neither LSD1 nor PHF8 was detected, but interestingly, we identified the histone demethylase JDJM1C/KDM3C (Fig. 4). Unlike LSD1 and PHF8, JMJD1C is a specific demethylase toward either active mark H3K9me1 or repressive mark H3K9me2, which participates in the progression of various tumors (90). As the net effect of JMJD1-mediated changes in H3K9 methylation status on gene transcription is unpredictable and may vary depending on the cellular context (*i.e.*, gene promoter, cell type, or physiological state), further investigation of the epigenetic role of JMJD1C as a NOTCH1 coactivator or corepressor may be required.

NICD protein stability, activity, and localization are tightly regulated by various posttranslational modifications including phosphorylation, methylation, hydroxylation, acetylation, and ubiquitinylation (7). The current NICD proteasomal degradation model demonstrates the importance of phosphorylation and ubiquitination at the C-terminal PEST domain site by the kinase CDK8 and the E3 ligase FBXW7, respectively (91, 92, 93). In addition, several studies showed that NICD protein stability is also regulated by deubiquitinating enzymes such as USP7 and USP8 in T-ALL and breast cancer (44, 45, 94). However, at sites outside the PEST domain, how posttranslational modifications affect NICD stability and turnover remains poorly understood (4). In this BioID study, various candidate protein modifiers and enzymes were identified (Table S3). As expected, CDK8 and FBXW7 were not recovered due to the lack of a PEST domain for our bait protein NICD. Remarkably, we identified several E3-type ligases and deubiquitinating enzymes, such as TRIM33, RBBP6, PIAS2, and USP34, which may affect Notch1 activity. It will be important to determine whether NICD is a substrate and how these interactions with E3 ligases and deubiquitinating enzymes affect NICD turnover. Notably, absence of the PEST domain and inactivation of FBW7 are common mechanisms for strong gain-of-function in NOTCH1 in human cancers such as T-ALL (8), breast cancer, and adenoid cystic carcinoma (9, 10, 11, 12). Therefore, another important question is whether deregulation of these interactions contributes to NICD tumorigenesis. Further characterization of these interactions may reveal mechanisms regulating NOTCH1 protein stability and turnover.

In the postgenomic and postproteomic era, one of the challenges is to study protein-protein functional interactions in living cells. We performed high-throughput BioID studies of the NOTCH1 nuclear interactome using two cell models, HEK293T and NIH3T3, which have unique advantages but also inherent limitations. On the one hand, we rationalized that low-level expression of endogenous Notch signaling in a background with a WT Notch1 genetic background might minimize interfering interactions from overexpressed NICD and its partners. Studies by many groups have shown that HEK293T and NIH3T3 cells naturally have lower Notch activity, making these two cell lines suitable for studying various components and mutants of Notch pathway function in various diseases, including noncancer conditions and cancers (95, 96, 97, 98, 99, 100, 101, 102). In our case, HEK293T and NIH3T3 are the model systems of choice for studying the Notch1 nuclear interactome using high-throughput methods such as BioID, with minimal interference from the endogenous expression of Notch1. On the other hand, we should be cautious in extrapolating our BioID results to other cell types without support from other experimental data, as Notch signaling may differ in noncancer and cancer cells (2, 7). Moreover, we fully agree and appreciate the future necessity of studying the nuclear interactome of NOTCH1 in the cell lines with NOTCH mutations. We emphasize here that additional efforts and manipulations may be required in further experiments such as BioID to avoid interference from endogenous mutant NICD highly expressed in NOTCH1-driven cell lines such as MOLT-4 and MB157 (8, 14, 103, 104).”

Since gain-of-function mutations in NOTCH1 can transform normal cells into tumor cells in many different cell types (7, 22, 23, 24), we rationalized our analysis to focus on common NOTCH1 interactors between cell types (Figs. 3 and 4). We also provided one proof-of-principle example for the first time in this study that GATAD2D and NOTCH1 may directly interact in NOTCH1-mutated MOLT-4 and MB157 cancer cells (Fig. 5, *B*–*E*). MOLT-4, an acute lymphoblastic leukemia cell line, possess a cis combination of the L1601PΔP heterodimerization mutation and PEST domain deletion and are highly sensitive to Notch and pan-HDAC inhibitors (103, 104). MB157 and MDA-MB-157 cell lines, both of which were derived from the same patient with triple-negative breast cancer, possess a NOTCH1 rearrangement associated with high levels of activated NOTCH1 protein and are also highly sensitive to Notch and pan-HDAC inhibitors (30, 105). To date, the role of GATAD2B in those NOTCH1-addicted cancers is unclear, although GATAD2B has been identified as a metastatic driver in lung cancer (106). In the future, it will be necessary to investigate the relative contribution of the common interactors such as GATAD2B to the cell type-specific and context-specific functions of NOTCH1. It also requires further investigation into how the cell type-specific interactors identified in this study contribute to cell type-specific mechanisms in the context of cancer cell survival and therapy.

In summary, our data support BioID as an excellent method to examine PPIs of NOTCH1 in living cells and demonstrate that it can serve as a powerful tool for probing the large multiprotein complexes that regulate chromatin structure and gene expression. We have identified a large set of nuclear proteins associated with NICD, including transcriptional factors, coactivators and corepressors, which are associated with many functional complexes. Furthermore, we found that NICD is associated with several protein modifiers and components of other signaling pathways that may affect Notch signaling and function. Importantly, biochemical and bioinformatic analyses led to the identification of multiple available drugs that may have therapeutic utility against Notch1-dependent cancers, although substantial research is needed to assess whether and how they affect Notch1 biological function. Together, the nuclear interactome of Notch1 oncoproteins discovered in this study in two different cell types should be a valuable resource for the field as we seek to uncover the mechanisms that fine-tune Notch signaling in tumorigenesis and provide therapeutic targets for Notch-addicted tumors.

## Experimental procedures

### Plasmids and cloning

All cloning was performed utilizing the In-Fusion Recombination system (Takara Bio). Empty pCW57.1 vector was a gift from David Root (Addgene #41393). To generate the negative control, myc-BioID2 was amplified by PCR from the previously generated myc-BioID2 pBabe puro (18) and inserted into the pCW57.1 vector using *Nhe*I and *Age*I restriction enzyme (RE) sites with an *EcoRI* RE site built into the reverse primer to allow for subsequent cloning. The mouse intracellular NICD fragment (amino acids 1749–2293, lacking the C-terminal PEST domain) was amplified by PCR from pBs-mNotch1-1C (gifted by Douglas Melton, Addgene #15079) and inserted into the BioID2-only pCW57.1 vector using *Eco*RI and *Age*I RE sites to make BioID2-NICD pCW57.1. The pRetroX-Tet3G system (Cat. No. 631188) was obtained from Takara Bio, Inc. Myc-BioID2 was PCR amplified from myc-BioID2 pBabe puro and inserted into pRetroX using the *BamHI* and *EcoRI* RE sites with a *NaeI* RE site built into the reverse primer to allow for subsequent cloning. NICD was PCR amplified from pBs-mNotch1-1C and inserted into BioID2-only pRetrox using the *NaeI* and *EcoRI* RE sites to make BioID2-NICD pRetroX.

### Cell culture and stable cell line generation

HEK293 Phoenix cells were obtained from National Gene Vector Biorepository. All other cell lines were obtained from the American Type Culture Collection (ATCC). MB157 (ATCC; CRL-7721), Phoenix, 293T (ATCC; CRL-3296), HEK293 (ATCC; CRL-1573), and NIH3T3 (ATCC; CRL1658) cells were cultured in Dulbecco's modified Eagle's medium with 4.5 g/L glucose, L-glutamine, and sodium pyruvate (Corning) and supplemented with 10% (v/v) fetal bovine serum (HyClone). SJSA-1 (ATCC; CRL-2098) cells were cultured in minimum essential medium-α medium (HyClone, SH30265FS) containing 10% fetal bovine serum (Thermo Fisher Scientific, ES009B) and 1% Penicillin-streptomycin (HyClone, SV30010). MOLT-4 (ATCC; CRL-1582) cells were grown in RPMI-1640 Medium (ATCC; 30–2001) and 10% fetal calf serum. All cells were maintained at 37 °C with a humidified atmosphere containing 5% CO2 and routinely tested for *mycoplasma* contamination. Stable cell lines were generated using lentiviral transduction (for HEK293, pCW57.1) with the ViraSafe Lentiviral Packaging System (Cell BioLabs, Inc; VPK-206) or retroviral transduction (for NIH3T3, pRetroX-Tet3G). Phoenix (retroviral) or 293T (lentiviral) cells were transfected with each construct using Lipofectamine 3000 (Thermo Fisher Scientific) according to the manufacturer's recommendations and incubated at 37 °C for 4 h. Then, the transfected cells were supplemented with fresh medium and further incubated at 37 °C for 72 h. Viral media was filtered through a 0.45 μm filter and added to HEK293 or NIH3T3 cells along with Polybrene (4 μg/ml; Santa Cruz Biotechnology). Forty-eight hours after transduction, puromycin (0.5 μg/ml; Thermo Fisher Scientific) or G418 (for 3T3 pRetroX system, 0.5 mg/ml; Corning) was added to target cells for 72 h (puromycin) or 7 days (G418), and viable cells were collected. Expression of BioID2-only and BioID2-NICD fusion proteins and functional biotinylation after addition of 50 μM biotin and 1 μg/ml doxycycline were further verified using IF and WB.

### Immunofluorescence

Cells grown on 1.5 mm glass coverslips were fixed with 3% (w/v) paraformaldehyde/phosphate-buffered saline for 10 min and permeabilized with 0.4% (w/v) Triton X −100/PBS for 15 min. To detect BioID2 fusion proteins, chicken anti-BioID2 (1:5000; BID2-CP-100; BioFront Technologies) was used (18). The anti-BioID2 antibody was detected using Alexa Fluor 568–conjugated goat anti-chicken (1:1000; A11041; Thermo Fisher Scientific). Alexa Fluor 488–conjugated streptavidin (1:1000; S32354; Thermo Fisher Scientific) was used to detect biotinylated proteins. DNA was detected with Hoechst dye 33342. Coverslips were mounted using 10% (wt/vol) Mowiol 4 to 88 (Polysciences). Epifluorescence images were obtained using a Nikon Eclipse NiE microscope (40 × /0.75 Plan Apo Nikon objective) with a charge-coupled device camera (CoolSnap HQ; Photometrics) linked to a workstation running NIS-Elements software (Nikon). All images were processed in Adobe Photoshop CC 2023 (https://www.techspot.com/downloads/6043-adobe-creative-cloud-photoshop.html) (Adobe) for cropping and brightness/contrast adjustment when applicable.

### WB analysis of biotinylated proteins

Total cell lysates (1.2 × 10^6^ cells) for WB were prepared in SDS–PAGE sample buffer, boiled for 5 min, and sonicated to shear DNA. Proteins were separated using 4 to 20% gradient gels (Mini-PROTEAN TGX; Bio-Rad) and transferred to nitrocellulose membrane (Bio-Rad). After blocking with 10% (vol/vol) adult bovine serum and 0.2% Triton X-100 in PBS for 30 min, the membrane was incubated with chicken anti-BioID2 primary antibody (1:5000; BID2-CP-100; BioFront Technologies). Anti-BioID2 primary antibody was detected using horseradish peroxidase–conjugated anti-chicken (1:40,000; A9046; Sigma-Aldrich) antibody. Biotinylated proteins were detected with horseradish peroxidase-conjugated streptavidin (1:40,000; ab7403; Abcam). The signals from antibodies were detected using enhanced chemiluminescence *via* a UVP BioImaging System (UVP).

### BioID pull-downs and digestion of biotinylated proteins

Large-scale BioID pull-downs were performed as previously described (17). Briefly, three biological replicates were performed for each cell line with distinct samples for each replicate (Fig. S2). For each large-scale BioID2 pull-down sample, two 10 cm dishes at 80% confluency were incubated with 1 μg/ml doxycycline for 24 h and then additionally supplemented with 50 μm biotin for 18 h. Cells were washed twice with PBS, lysed in 8 M urea 50 mM Tris pH 7.4 containing protease inhibitor (87785: Thermo Fisher Scientific) and DTT, incubated with a universal nuclease (88700: Thermo Fisher Scientific), and sonicated to further shear DNA. Lysates were precleared with Gelatin Sepharose 4B beads (17095601; GE HealthCare) for 2 h and then incubated with Streptavidin Sepharose High Performance beads (17511301: GE HealthCare) for 4 h. Streptavidin beads were washed four times with 8 M urea 50 mM Tris pH 7.4. Ten percent of the beads were collected for WB analysis and the other 90% were resuspended in 50 mM ammonium bicarbonate containing 1 mM biotin for MS analysis.

Beads were thawed and resuspended with 8 M urea, 50 mM ammonium bicarbonate, and cysteine disulfide bonds were reduced with 10 mM tris (2-carboxyethyl) phosphine (TCEP) at 30 °C for 60 min. Cysteines were alkylated with 30 mM iodoacetamide for 30 min at room temperature in the dark. After alkylation, urea was diluted to 1 M urea and proteins were digested overnight with a MS-grade Trypsin/Lys-C mix (Promega). Finally, the beads were pulled down and the peptide-containing solution was collected into a new tube. The beads were then washed once with 50 mM ammonium bicarbonate to increase peptide recovery. After digestion, samples were acidified with formic acid (FA) and subsequently desalted using an AssayMap C18 cartridges mounted on an Agilent AssayMap BRAVO Liquid Handling System (Agilent). Briefly, C18 cartridges were conditioned first with 100% acetonitrile (ACN) and then with 0.1% FA. Samples were then loaded onto conditioned C18 columns, washed with 0.1% FA, and eluted with 60% ACN, 0.1% FA. Finally, organic solvents were removed in a SpeedVac concentrator prior to LC-MS/MS analysis.

### Liquid chromatography and mass spectrometry (LS-MS) assay

Dried samples were reconstituted with 2% ACN-0.1% FA and quantified by NanoDrop spectrophometer (Thermo Fisher Scientific) prior to LC-MS/MS analysis using a Proxeon EASY nanoLC system (Thermo Fisher Scientific) coupled to an Orbitrap Elite mass spectrometer (Thermo Fisher Scientific). Peptides were separated using an analytical C18 Acclaim PepMap column (75 μm x 250 mm, 2 μm particles; Thermo Fisher Scientific) using a 117-min gradient, at a flow rate of 300 μl/min, consisting in: 1% to 6% B in 1 min, 6% to 23% B in 72 min, 23% to 34% B in 45 min, 34% to 48% B in 2 min, and 48% to 98% B in 2 min (A = FA, 0.1%; B = 80% ACN: 0.1% FA). The MS was operated in positive data-dependent acquisition mode. MS1 spectra were measured in the Orbitrap with a resolution of 60,000 (AGC target: 3e4; maximum injection time: 100 ms; mass range: from 375 to 1400 m/z). After the survey scan, up to 10 of the most intense precursors ions were fragmented by CID in the ion trap cell (Isolation window: 2 m/z; charge state: + 2; normalized collision energy: 35%). Resulting fragments were detected in the Ion trap cell with a rapid scan (AGC target: 1e4; maximum injection time: 100 ms). Precursor dynamic exclusion was set to 30s, with a 10 ppm mass tolerance around the precursor.

### MS data analysis

All mass spectra from were analyzed with MaxQuant software (https://www.maxquant.org/) version 1.5.5.1. MS/MS spectra were searched against the *Homo sapiens* Uniprot protein sequence database (version January 2018) and GPM cRAP sequences (commonly known protein contaminants). Precursor mass tolerance was set to 20 ppm and 4.5 ppm for the first search where initial mass recalibration was completed and for the main search, respectively. Product ions were searched with a mass tolerance of 0.5 Da. The maximum precursor ion charge state used for searching was 7. Carbamidomethylation of cysteines was searched as a fixed modification, while oxidation of methionines and acetylation of protein N-terminal were searched as variable modifications. Enzyme was set to trypsin in a specific mode and a maximum of two missed cleavages was allowed for searching. The target-decoy-based false discovery rate filter for spectrum and protein identification was set to 1%. Common background proteins were removed, including keratins, tubulins, histones, and ribosomal proteins (107). Proteins were classified as candidate interactors if they were identified in at least two of three triplicate samples and label-free quantification intensities were at least 3-fold greater compared to control (16, 108, 109).

### Functional and network analyses of significant interactors

For functional annotation analysis, pathways, diseases, drug targets, and gene ontology analyses were performed by IPA (http://www.ingenuity.com) with default parameters. Candidate interactors were converted to official gene symbol and then selected as the identifier in this tool. *H. sapiens* was selected as the species of origin. Assignment of previously identified NOTCH1 interactors was based on annotation in the BioGRID database (https://thebiogrid.org/) (27). Using the Broad Institute's CLUE Drug Repurposing Hub database (version 4/3/2023, https://www.broadinstitute.org/drug-repurpose-hub), we annotated those known compounds or drugs in the database to target our candidate Notch1 protein interactors.

For network analysis, The STRING software (www.string-db.org) was utilized for detecting protein interaction clusters, complexes, and subnetworks (accessed on 12 April 2023) (110). The betweenness centrality values were calculated using the NetworkAnalyzer Cytoscape plugin (48). Significant interactors were input using symbol ID. Organism was set to *H. sapiens*. Networks were visualized using either default or yFiles Radial Layout in Cytoscape (v3.8.2) (111).

### Proximity-based ligation assay

PLA was performed according to the manufacturer’s instructions (Sigma-Aldrich) (112, 113). Briefly, a total of 1 × 10^4^ SJSA-1 or 293T cells were seeded overnight onto a 4-well glass chamber slide (Thermo Fisher Scientific). Cells were fixed with 4% paraformaldehyde in PBS for 10 min. Cells were then permeabilized and blocked with blocking solution for 60 min, and probed overnight in humidified chamber at 37 °C with antibodies directed against Notch1 (C-20, sc-6014, Santa Cruz Biotechnology) and/or HDAC1 (10E2, sc-81598, Santa Cruz Biotechnology). Cells were then treated with Duolink *In Situ* Red Starter Mouse/Rabbit kit (Sigma-Aldrich) as per manufacturer’s instructions. Cells were then washed, and the nuclei were stained with 4′,6-diamidino-2-phenylindole (DAPI). Images of the nuclei, proximity ligated foci were acquired using Z-stack images captured with an Olympus FV300 upright confocal microscope (Olympus). Images were cropped using ImageJ software (https://imagej.net/ij/download.html) (National Institutes of Health). The fluorescence intensity of proximity ligated foci was measured by ImageJ software using 8 bit images. Briefly, the tracing tool was used to outline individual cells, and the “measure” function was used to obtain mean fluorescence intensity of each proximity ligated foci. Total fluorescence intensity in each cell was normalized by cell area and plotted as a dot in the graph. Statistical analysis of data was done by unpaired Student’s two-sided *t* test.

### *In vivo* cross-linking, endogenous Co-IP, and immunoblotting

*In vivo* cross-linking procedures to isolate weak protein complex have been described previously in detail (51, 52, 53). Briefly cells were washed once in prewarmed (37 °C) PBS and incubated with 1 mM DSP (D3669, Sigma-Aldrich) in PBS at 37 °C for 10 min in a CO_2_ incubator. The reaction was quenched with 20 mM Tris in PBS at room temperature for 10 min. After the cross-linking, nuclear protein extraction and Co-IP procedures were performed using the Nuclear Complex Co-IP Kit (54001, Active Motif) according to the manufacturer's instructions. Protein concentration was determined using the Pierce BCA protein assay kit (23225, Thermo Fisher Scientific). Nuclear extracts (250–500 μg) were immunoprecipitated using 1 to 5 μg of NOTCH1 antibody (sc-6014, Santa Cruz) and normal IgG control antibody (31235, Thermo Fisher Scientific). Co-IP proteins were resolved on 7.5% SDS-PAGE gels (4561024, Bio-Rad) and analyzed by a previously described WB system (83) using primary antibodies against GATAD2B (1:1000, PA5-53536, Thermo Fisher Scientific), or RBPJ (1:500, sc-271128, Santa Cruz), or DDX15 (1:500, sc-271686, Santa Cruz). The membranes were incubated with secondary antibodies StarBright Blue 700 Goat Anti-Rabbit IgG (1:2,500, 12004162, Bio-Rad) and StarBright Blue 520 Goat Anti-Mouse IgG (1:2,500, 12005867, Bio-Rad). Signals from Blots were measured using a ChemiDoc MP Imaging System (Bio-Rad). Chameleon Duo Pre-Stained Protein Ladder (928–60000, Li-Cor Biosciences) was used as a molecular weight marker.

## Data availability

All relevant data are contained within this research article and in the supporting information.

## Supporting information

This article contains supporting information (14).

## Conflicts of interest

Sanford Research has licensed BioID reagents developed by K. J. R. to BioFront Technologies. The remaining authors declare that they have no conflicts of interest with the contents of this article.

## Supporting information

Supplemental Figures Figure S1. **Workflow comparison of the BioID method with tandem affinity purification coupled with mass spectrometry (TAP-MS).** (*Left*) In BioID, NICD was fused to a mutant biotin ligase (BioID2), which biotinylates (biotin, *red dot*) lysine residues on proximate proteins. Following vigorous cell lysis, the biotinylated proteins were isolated using streptavidin-conjugated beads. After extensive washing, the peptides were eluted from the solid support by tryptic proteolysis and identified using quantitative mass spectrometry (MS). (*Right*) Target protein (NICD) fused in-frame with two epitope tags including Flag and HA in a TAP-MS study of SupT1 cells (14). Cells were disrupted using mild lysis conditions to maintain protein-protein interactions. Two-step affinity purification was used to isolate the tagged "bait" protein and its binding partner. After gentle washing, proteins were eluted, converted to peptides and identified using MS. For both methods, candidate interacting proteins can be further explored by bioinformatic analysis and other methods such as co-immunoprecipitation (Co-IP) and proximity ligation assays (PLA). Figure S2. **Outline of the BioID studies in HEK293 and 3T3 cells.** Doxycycline inducible BioID2-only or BioID2-NICD plasmids were stably integrated into HEK293 or 3T3 cells. Expression was induced with doxycycline overnight, followed by addition of 50 μM biotin to induce biotinylation in biological triplicates with two 10-cm plates of cells pers ample. Cells were lysed under stringent conditions and biotinylated proteins were collected on streptavidin-conjugated beads for subsequent identification *via* MS analysis. Figure S3. **STRING protein-protein interaction (PPI) network analyses of NICD core-proximal proteins reveal their significant association.** The network contains 83 nodes with 250 edges (vs. 78 expected edges); average node degree 6.02; clustering coefficient 0.558; enrichment p-value < 1.0e-16. Figure S4. ***In vivo* interactions between endogenous proteins in non-cancer cells and Notch-addicted cancer cells.***A*, *Validation of the NOTCH1-HDAC1 interaction in human 293T cells by the proximity ligation assay.* Representative images of PLA signal and nuclear staining (DAPI) showing cells probed with anti-Notch1 alone (*top row*), anti-HDAC1 alone (*middle row*) or both antibodies (*bottom row*). The scale bars at low magnification (*two left panels*) represent 100 μm and the scale bars with high magnification (*two right middle panels*) represent 20 μm. Far right panel shows quantification of mean PLA per foci per cell, with standard deviation; n = 3. *B*, Western blot (WB) analysis of immunoprecipitates (IP: NOTCH1 and control IgG antibodies) and nuclear extracts from MOLT-4 leukemia cells cross-linked with DSP showing endogenous protein RBPJ and DDX15 co-immunoprecipitated with NOTCH1 antibody. Immunoprecipitating antibody was raised in rabbits and immunoblotting antibodies were raised in mice. *Arrow heads* indicate RBPJ and DDX15. The ladder represents the molecular weight (kDa) of the protein size. The Input Lane represents the initial input in the experiment (1.5%). *C*, WB analysis of immunoprecipitates (IP: NOTCH1 and control IgG antibodies) and nuclear extracts from MB157 breast cancer cells cross-linked with DSP showing that the band for the endogenous protein RBPJ was easily detected, but the band for endogenous DDX15 was very faint. The input lanes represent the initial input in the experiment (5% and 2%, respectively). Figure S5. **STRING network of NICD core-proximal interactors and potential drug targets.** STRING network analysis shows that 10 core-proximal interactors of NICD, which can be targeted by currently available drugs or agents that are FDA-approved or examined in clinical trials or preclinical studies.

Table S1 (Related to Fig. 1): NOTCH1 proximal and interacting proteins identified by MS analysis in HEK293 cells. *A*, MS data for samples 530-535 in HEK293 cells. Biological triplicate for BioID2-only (samples 530, 531, 532) and BioID2-NICD (samples 533, 534, 535). *B*, analysis of samples 530-535. C. BioID candidate interactors of Notch1 in HEK293 cells.

Table S2 (Related to Fig. 2): NOTCH1 proximal and interacting proteins identified by MS analysis in 3T3 cells. *A*, MS data for samples 536-541 in 3T3 cells. Biological triplicate for BioID2-only (samples 536, 537, 538) and BioID2-NICD (samples 539, 540, 541). *B*, analysis of samples 536-541. *C*, BioID candidate interactors of Notch1 in 3T3 cells.

Table S3 (Related to Fig. 3): Common NOTCH1 proximal and interacting proteins identified in HEK293 and 3T3 cells. *A*, common interactors between two of the three groups (HEK293, 3T3 and BioGRID). (Related to Fig. 3*A*). *B*, functional Annotation of common candidate interactors for cellular components and molecular functions using Ingenuity Pathway Analysis (IPA). (Related to Fig. 3, *B* and *C*). *C*, pathways analysis of common candidate interactors of Notch1 (Related to Fig. 3*D*).

Table S4 (Related to Fig. 4): Network analysis of NICD core-proximal proteins. *A*, interaction network analysis of common candidate interactors of Notch1 using STRING software. *B*, curated classes of NICD proximal proteins based on STRING analysis.

Table S5 (Related to Fig. 5): Potential drug targets revealed by the Notch1 BioID interactome study. *A*, centrality analysis of NICD proximal interacting proteins using the NetworkAnalyzer Cytoscape plugin. *B*, potential drug targets and drugs identified in CLUE drug library. *C*, potential drug targets and drugs identified in Ingenuity Pathway Analysis (IPA).
