# Supplementary material for: Comprehensive analysis of the proximity-dependent nuclear interactome for the oncoprotein NOTCH1 in live cells
Source: J Biol Chem. 2023 Dec 1;300(1):105522. doi: 10.1016/j.jbc.2023.105522 (PMC10788534; doi:10.1016/j.jbc.2023.105522)
Supplement: Supplemental Figures — Figure S1. Workflow comparison of the BioID method with tandem affinity purification coupled with mass spectrometry (TAP-MS). (Left) In BioID, NICD was fused to a mutant biotin ligase (BioID2), which biotinylates (biotin, red dot) lysine residues on proximate proteins. Following vigorous cell lysis, the biotinylated proteins were isolated using streptavidin-conjugated beads. After extensive washing, the peptides were eluted from the solid support by tryptic proteolysis and identified using quantitative mass spectrometry (MS). (Right) Target protein (NICD) fused in-frame with two epitope tags including Flag and HA in a TAP-MS study of SupT1 cells (14). Cells were disrupted using mild lysis conditions to maintain protein-protein interactions. Two-step affinity purification was used to isolate the tagged "bait" protein and its binding partner. After gentle washing, proteins were eluted, converted to peptides and identified using MS. For both methods, candidate interacting proteins can be further explored by bioinformatic analysis and other methods such as co-immunoprecipitation (Co-IP) and proximity ligation assays (PLA). Figure S2. Outline of the BioID studies in HEK293 and 3T3 cells. Doxycycline inducible BioID2-only or BioID2-NICD plasmids were stably integrated into HEK293 or 3T3 cells. Expression was induced with doxycycline overnight, followed by addition of 50 μM biotin to induce biotinylation in biological triplicates with two 10-cm plates of cells pers ample. Cells were lysed under stringent conditions and biotinylated proteins were collected on streptavidin-conjugated beads for subsequent identification via MS analysis. Figure S3. STRING protein-protein interaction (PPI) network analyses of NICD core-proximal proteins reveal their significant association. The network contains 83 nodes with 250 edges (vs. 78 expected edges); average node degree 6.02; clustering coefficient 0.558; enrichment p-value < 1.0e-16. Figure S4. In vivo interactions between endogenous [file mmc1.pdf]

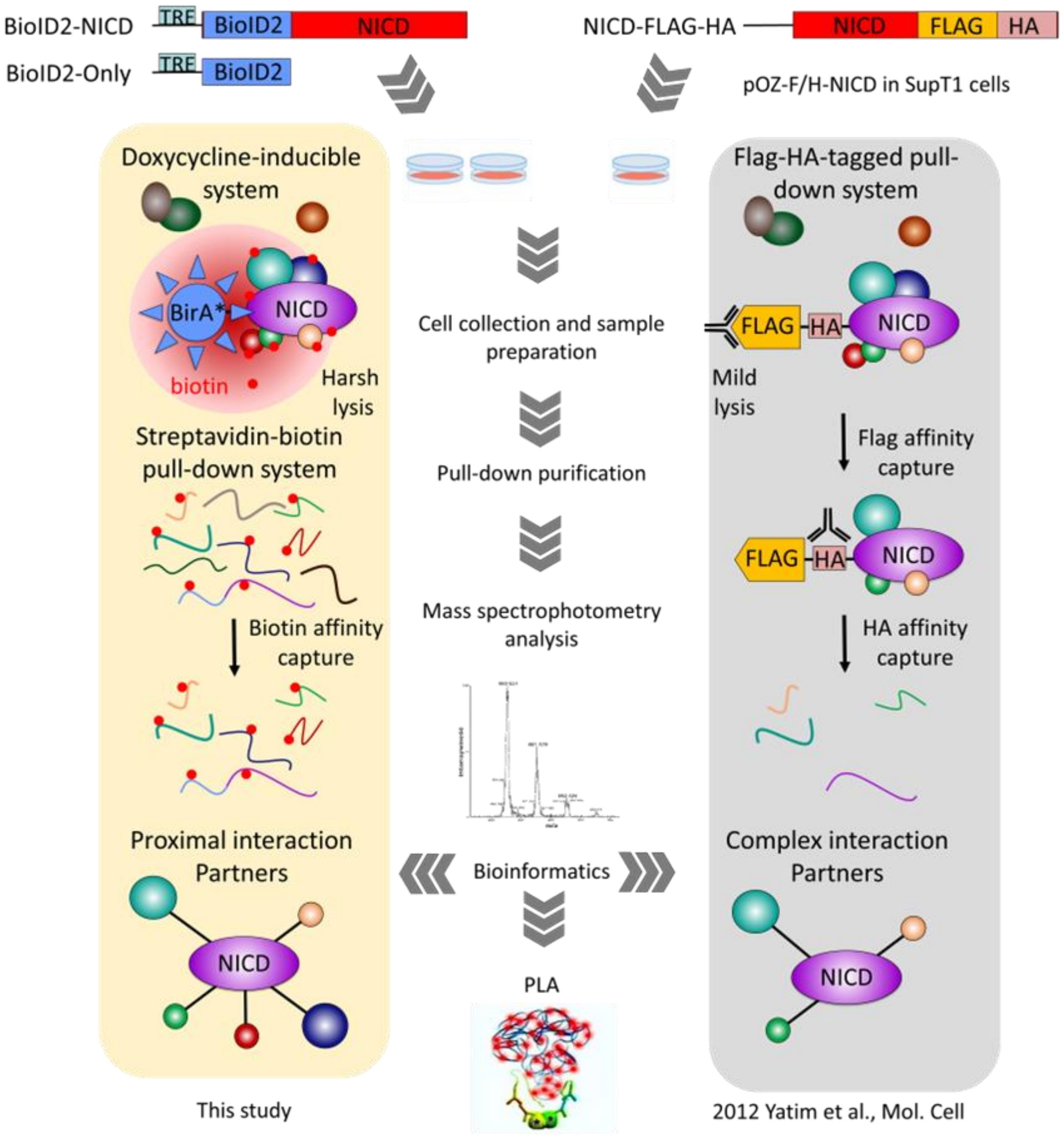
BioID2-NICD
TRE
BioID2
NICD
BioID2-Only
TRE
BioID2
NICD-FLAG-HA
NICD
FLAG
HA
pOZ-F/H-NICD in SupT1 cells
Doxycycline-inducible system
BirA*
NICD
biotin
Harsh lysis
Streptavidin-biotin pull-down system
Biotin affinity capture
Proximal interaction Partners
NICD
This study
Cell collection and sample preparation
Pull-down purification
Mass spectrophotometry analysis
Bioinformatics
PLA
Flag-HA-tagged pull-down system
FLAG
HA
NICD
Mild lysis
Flag affinity capture
FLAG
HA
NICD
HA affinity capture
Complex interaction Partners
NICD
2012 Yatim et al., Mol. Cell

Figure S1

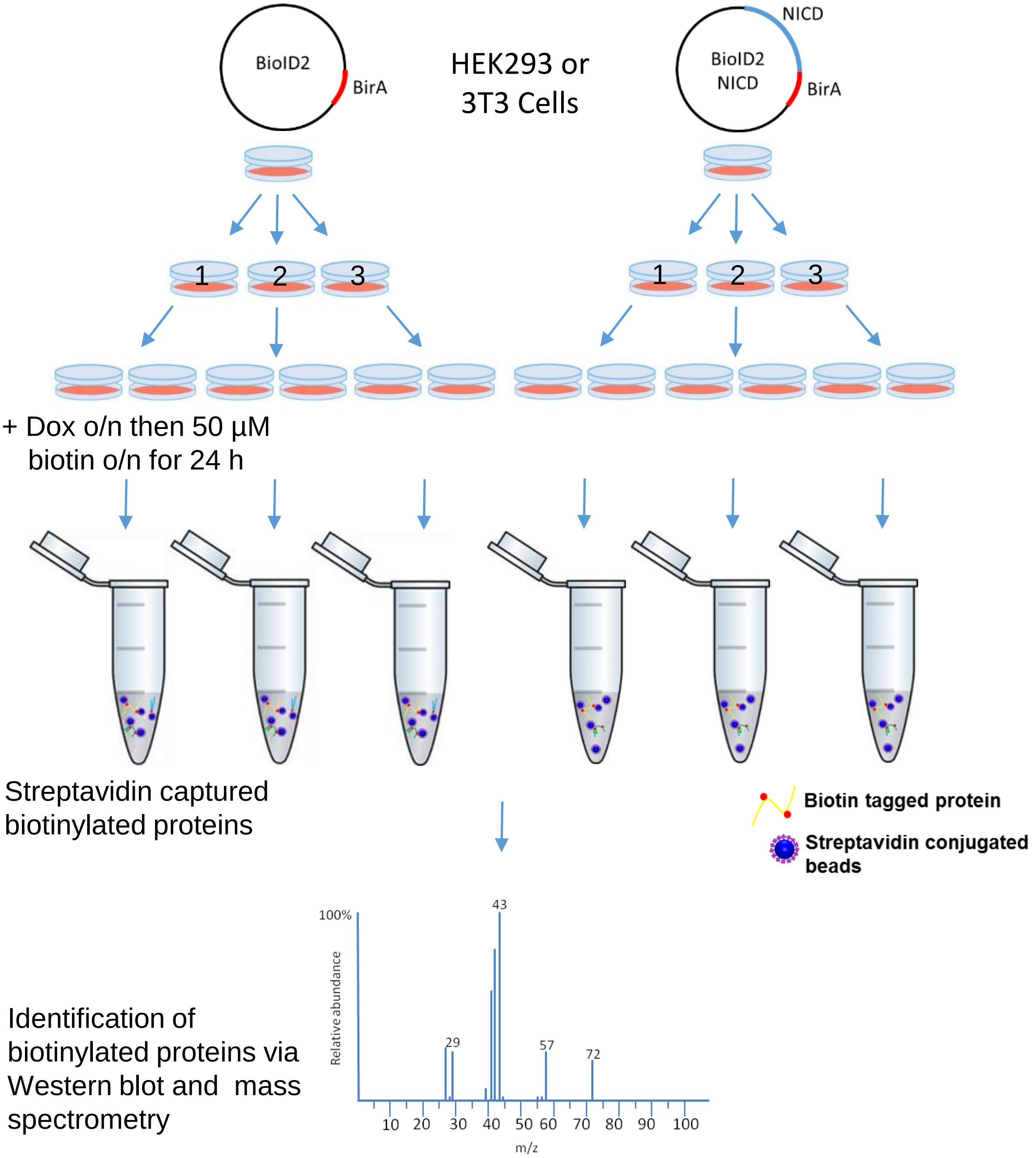
BioID2
BirA
HEK293 or
3T3 Cells
NICD
BioID2
NICD
BirA
1
2
3
1
2
3
+ Dox o/n then 50 µM
biotin o/n for 24 h
Streptavidin captured
biotinylated proteins
Biotin tagged protein
Streptavidin conjugated
beads
Identification of
biotinylated proteins via
Western blot and mass
spectrometry
100%
Relative abundance
43
29
57
72
10 20 30 40 50 60 70 80 90 100
m/z

Figure S2

A

number of nodes: 83
number of edges: 250
average node degree: 6.02
avg. local clustering coefficient: 0.558
expected number of edges: 78
PPI enrichment p-value: < 1.0e-16

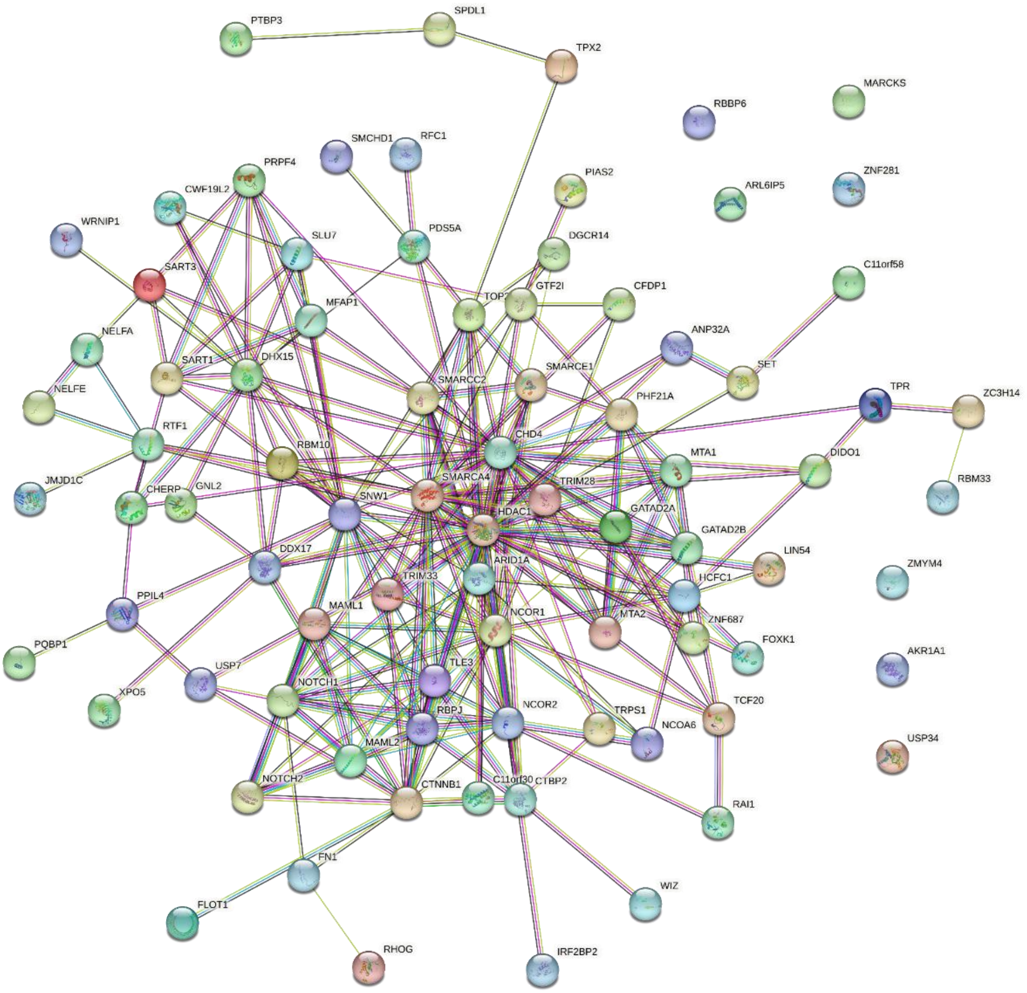

Figure S3

A Proximity Ligation Assay (PLA) – 293T cells

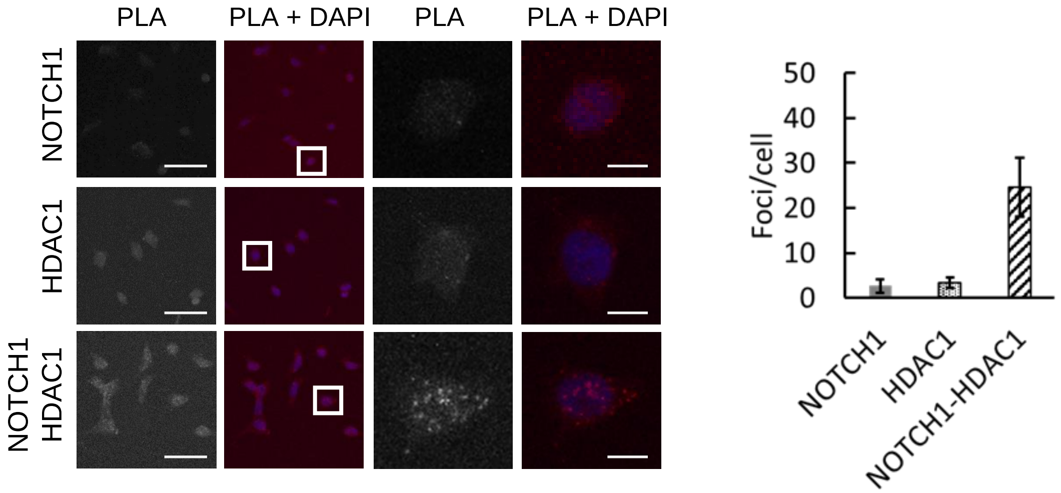

B *In vivo* crosslinking and co-immunoprecipitation (Co-IP)

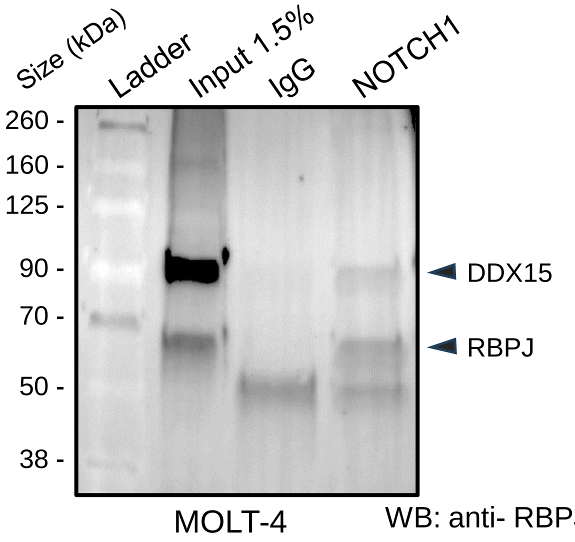

C

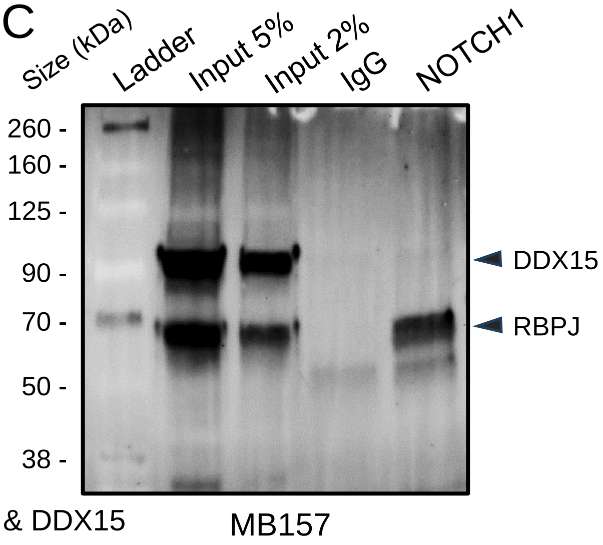

WB: anti- RBPJ & DDX15

Figure S4

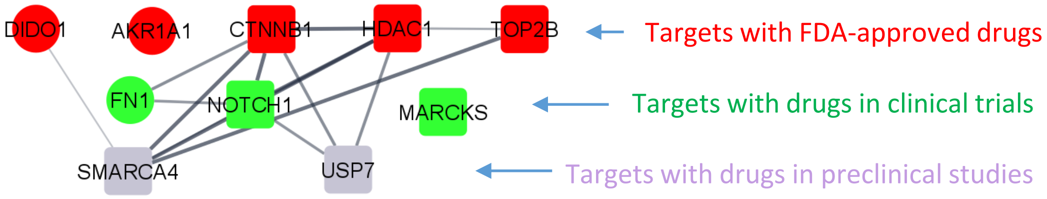
DIDO1
AKR1A1
CTNNB1
HDAC1
TOP2B
FN1
NOTCH1
MARCKS
SMARCA4
USP7
Targets with FDA-approved drugs
Targets with drugs in clinical trials
Targets with drugs in preclinical studies

Figure S5
